# Utilization of large language models in decision-making for sustainability in radiology

**DOI:** 10.3389/fmed.2025.1632925

**Published:** 2025-09-30

**Authors:** Viktoria Palm, Patricia Leutz-Schmidt, René Michael Mathy, Benedikt Jakob Schwaiger, Hans-Ulrich Kauczor, Hyungseok Jang, Sam Sedaghat

**Affiliations:** ^1^Clinic for Diagnostic and Interventional Radiology (DIR), Heidelberg University Hospital, Heidelberg, Germany; ^2^Translational Lung Research Center (TLRC), German Center for Lung Research (DZL), University of Heidelberg, Heidelberg, Germany; ^3^Diagnostic and Interventional Radiology with Nuclear Medicine, Thoraxklinik Heidelberg, Heidelberg, Germany; ^4^Department of Neuroradiology, School of Medicine and Health, Technical University of Munich, Munich, Germany; ^5^Department of Radiology, University of California, Davis, Davis, CA, United States

**Keywords:** large language model, sustainability, green radiology, ChatGPT, radiology

## Abstract

**Introduction:**

Radiology has a significant environmental impact, but guidance on how to effectively implement sustainable practices in this field is limited. This study investigated the performance of large language models (LLMs) in providing sustainability advice for radiology.

**Methods:**

Four state-of-the-art LLMs, namely ChatGPT-4.0 (CGT), Claude 3.5 Sonnet (CS), Gemini Advanced (GA), and Meta Llama 3.1 405b (ML), were evaluated based on their answers to 30 standardized questions covering sustainability topics such as energy consumption, waste management, digitalization, best practices, and carbon footprint. Three experienced readers rated their response for quality (OQS), understandability (US), and implementability (IS) using a 4-point scale. A mean quality score (MQS) was derived from these three attributes.

**Results:**

The overall intraclass correlation was good (ICC = 0.702). Across the 30 questions on sustainability in radiology, all four LLMs showed good to very good performances, with the highest ratings being achieved in understandability (CGT/GA/ML 3.91 ± 0.29; CS 3.99 ± 0.11), underlining the excellent language skills of these models. CS emerged as the top performer across most topics, with an MQS of 3.95 ± 0.22, frequently achieving the highest scores. ML showed the second highest performance with an MQS of 3.84 ± 0.37, followed by CGT with an MQS of 3.78 ± 0.42 and GA with an MQS of 3.73 ± 0.44. Accordingly, CGT and GA showed comparable results, while GA consistently received lower mean scores than the other LLMs. None of the LLMs provided answers that were rated insufficient.

**Conclusion:**

Our findings highlight the potential of LLMs such as Claude 3.5 Sonnet, ChatGPT-4.0, Meta Llama 3.1, and Gemini Advanced to advance sustainable practices in radiology, with thoughtful model selection further enhancing their positive impact due to model variations.

## Introduction

The healthcare sector’s environmental impact has garnered increasing attention, with radiology emerging as a particularly resource-intensive specialty. Radiological practices rely heavily on advanced imaging technologies, thereby significantly contributing to energy consumption, waste production, and carbon emissions (1, 2). Radiological devices such as CT and MRI scanners are significant energy consumers, with each unit contributing up to 26,226 kWh and 134,037 kWh per unit annually, respectively (3). Energy use for cooling systems alone contributed 492,624 kWh across multiple scanners, representing 44.5% of the total energy consumption. Furthermore, the overall energy consumption of MRI and CT scanners is predominantly driven by the idle periods rather than the short, high-energy peaks during image acquisition (3–5). Between scans, continuous power is required to maintain system readiness, cooling mechanisms, and, in the case of MRI, superconducting magnet stability, resulting in significantly higher cumulative energy demand in standby mode compared to active scanning. Hence, in MRI, the unproductive idle status consumes up to four times more energy than during imaging acquisition, accounting for 72–91% of total MRI energy consumption (4, 6, 7). Given that radiology is responsible for a considerable portion of a hospital’s overall energy usage, there is an urgent need to implement more sustainable practices within this field (8, 9). Beyond energy consumption, another key sustainability challenge in MRI is its reliance on helium, a non-renewable resource essential for cooling superconducting magnets. As the largest helium consumer, the healthcare sector is responsible for one-third of worldwide resource consumption (10). Sustainable aspects, resource shortages, and increasing costs have raised concerns about MRI-associated helium consumption, prompting the development of conservation strategies such as zero-boil-off systems and helium recycling technologies (11). One example of such innovation is Philips’ BlueSeal magnet technology, which has already saved more than 1,000,000 L of helium since 2018, with nearly 600 units installed globally (12). Implementing these measures is crucial for reducing helium waste in an environmentally responsible manner. Waste management and the use of contrast agents in radiology also present critical concerns due to their substantial environmental and health impacts, with waste and water consumption contributing up to 5% of the healthcare sector’s CO2-equivalent emissions (13). Studies indicate that a significant portion of medical waste in radiology is generated by single-use materials such as syringes, contrast agent vials, and protective covers, contributing to the overall healthcare waste burden (13, 14). Contrast agents, particularly iodinated contrast media (ICM) used in CT scans, pose environmental risks when excreted by patients and enter wastewater systems, where they can persist and potentially impact aquatic ecosystems (15, 16). Strategies to reduce contrast agent waste, such as dose optimization and closed-loop contrast delivery systems, are being explored to minimize environmental impact while maintaining diagnostic efficacy (7, 15, 17). Thus, sustainability in radiology encompasses various critical issues, including reducing energy consumption, waste management, and minimizing the carbon footprint associated with imaging procedures.

Additionally, digitalization and teleradiology have significantly transformed radiological practice, leading to increased efficiency, improved diagnostic accuracy, and enhanced accessibility to imaging services. Studies show that adopting digital radiology systems can reduce report turnaround time by up to 50%, enabling faster diagnoses (18, 19). In teleradiology, the remote interpretation of imaging studies has expanded rapidly, resulting in more efficient workload distribution, increased radiologist productivity, and the ability to provide 24/7 coverage for urgent cases (20, 21). Digital solutions, particularly artificial intelligence (AI), are advancing diagnostic accuracy significantly. In a recent study, AI-assisted detection rates for lung nodules in radiographs reached 0.59%, compared to 0.25% in non-AI-supported diagnoses, showing increased lung nodule detection (22). These advances highlight the critical role of digital solutions in optimizing radiology workflows and expanding access to care through teleradiology. With the advent of large language models (LLMs), there is growing interest in leveraging these advanced AI tools to provide expert advice across various domains, including sustainability in radiology (23–26). A nationwide survey in Germany on sustainability in radiology revealed that 16% of employees and employers state that qualifications for implementing sustainable measures in radiology are one of the major obstacles (7). LLMs such as ChatGPT, Claude, Gemini, and Meta AI are designed to process and generate human-like text based on vast datasets, making them potentially valuable resources for radiologists seeking guidance on sustainable practices. However, the effectiveness of these models in delivering accurate, practical, and relevant advice specific to sustainability in radiology remains largely unexplored. Given the unique challenges and sustainability requirements in radiology, assessing if and how these LLMs can support healthcare professionals in implementing environmentally responsible practices is imperative. Therefore, the primary objective of this study is to evaluate the performance of various LLMs in offering sustainability advice in radiology. Specifically, this study will compare the capabilities of ChatGPT 4.0 (CGT), Claude 3.5 Sonnet (CS), Gemini Advanced (GA), and Meta AI based on Llama 3.1 405b (ML) in addressing key sustainability topics relevant to radiologists.

## Materials and methods

### LLMs and questions

This study presents a comparative analysis of four state-of-the-art large language models (LLMs) to evaluate their performance across key dimensions of sustainability in radiology. The selected LLMs included the following: ChatGPT-4.0 (OpenAI, United States), Gemini Advanced (Google, United States), Meta AI based on Llama 3.1 405B (Meta AI, United States), and Claude 3.5 Sonnet (Anthropic, United States). Each model was prompted with an identical set of 30 standardized questions designed to assess its capabilities in addressing sustainability-related challenges and concepts within the field of radiology.

The development of these 30 questions followed a structured, multi-source approach. Questions were derived from a combination of current literature, professional guidelines, search engine (Google), and LLM queries. This ensured a broad and representative coverage of relevant topics. The initial draft of questions was rigorously designed and reviewed by four board-certified radiologists, each with more than 5 years of experience and specialized knowledge in sustainable healthcare practices. Their expertise was instrumental in ensuring the questions’ clinical relevance, evidence-based accuracy, and thematic coherence.

Through a consensus-driven process, the reviewers finalized the question set, making appropriate modifications and categorizing the questions into five key thematic areas:

1. Radiological Devices.
2. Waste Management.
3. Digitalization.
4. Practices and Policies.
5. Environmental Impact.

Each category contained six refined, focused questions, resulting in a total of 30 comprehensive prompts that reflect critical issues in sustainable radiology (see Supplementary material). A final manual re-review validated the question set for completeness and alignment with current best practices. These standardized prompts were then submitted to each LLM to ensure a consistent basis for comparative evaluation. We performed a first-input scheme for that part of the study as this is the most realistic real-world scenario.

### Analysis from radiologists

The responses from the chatbots were assessed using three customized scoring systems: (1) Overall Quality Score (OQS), (2) Understandability Score (US), and (3) Implementability Score (IS) (27, 28). The average of these three scores was plotted to a Mean Quality Score (MQS). The OQS represents the accuracy, completeness, and relevance of the responses. We evaluated clarity and ease of understanding, while the IS assessed the practicality and applicability of the recommendations. Each pattern was rated on a four-point scale: 1 – insufficient, 2 – moderate, 3 – good, and 4 – very good (28). Responses rated as insufficient lacked key information, were poorly structured, were difficult to understand, or were impractical to implement. Moderate responses contained some relevant information but were marked by noticeable gaps, issues with clarity, or difficulties in implementation. Good responses were largely coherent, distinct, mostly complete, and practical, with only minor shortcomings. Very good responses were comprehensive, precise, well-organized, and easy to implement. For example, an IS score of 4 implies a recommendation that can be directly implemented in radiological workflow without further modification, such as “adopting energy-efficient imaging devices with standby power reduction modes.”

**Figure 1:**
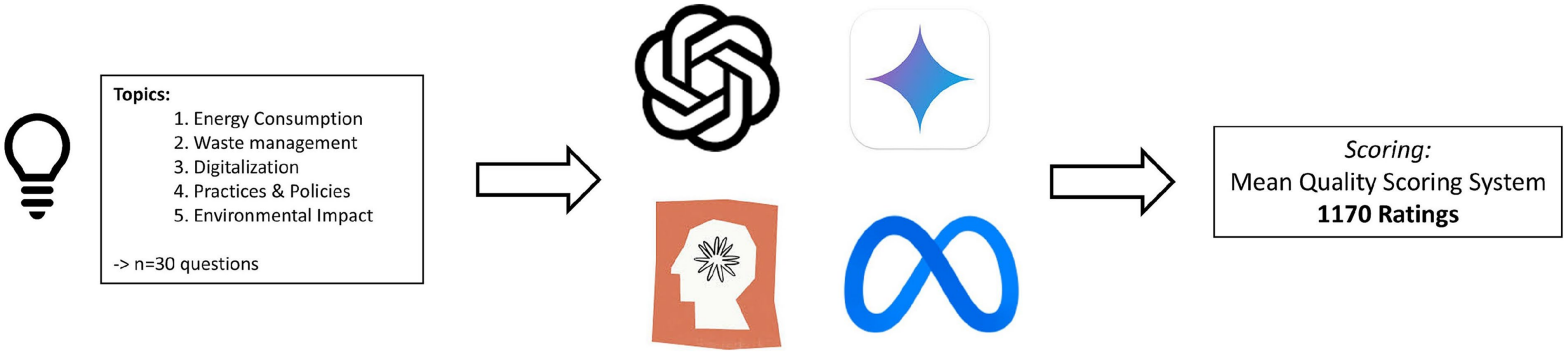
Flowchart of study design.

Three senior radiologists with expertise in greening radiology and sustainability management independently reviewed each of the 30 responses from the four chatbots using this scoring system, resulting in 1170 ratings (Figure 1). The performance of each LLM version was evaluated for each individual question, with a consensus established to determine superiority.

### Attributes of the scoring system

I. Overall Quality Score (OQS; evaluation of answer content by accuracy, completeness, and relevance) comprising the following points:

1. Insufficient: The answer is incomplete, incorrect, or does not address the question adequately.
2. Moderate: The answer addresses the question but is missing some key points or has some inaccuracies.
3. Good: The answer is accurate and covers most critical points with minor omissions.
4. Very Good: The answer is thorough, accurate, and covers all key points effectively.

II. Understandability Score (US; assessing LLM answer by clarity and readability) comprising the following points:

1. Insufficient: The answer is confusing, poorly organized, or difficult to read.
2. Moderate: The answer is somewhat clear but has issues with organization or language that affect understanding.
3. Good: The answer is clear and easy to understand, with minor issues.
4. Very Good: The answer is very clear, well-organized, and easy to read.

III. Implementability Score (IS; practical applicability of recommendations) comprising the following points:

1. Insufficient: The recommendations are impractical, unrealistic, or too vague to implement.
2. Moderate: Some recommendations are practical, but others are vague or unrealistic.
3. Good: The recommendations are practical and mostly feasible, with minor adjustments needed.
4. Very Good: The recommendations are highly practical, feasible, and easily implemented.

### Statistics

Data were presented as mean values [with standard deviation (SD)], numerical counts, or percentages. Descriptive statistics were used where appropriate. Intraclass correlation was assessed by calculating the intraclass correlation coefficient (ICC). The ICC was categorized as moderate (0.41–0.6), good (0.61–0.8), or excellent (0.81–1.0). The Kruskal-Wallis test was used to identify differences between the groups. A post-hoc Mann–Whitney-U-Test was performed to determine the significant groups separately. Statistical significance was set at a *p*-value of < 0.05. The *p*-values indicate whether there are overall differences between the LLMs and differences within each subcategory. Analyses were conducted using IBM-SPSS version 28.0 (IBM, Armonk, NY, United States), and Python 3.12 (Python Software Foundation) was used to generate charts.

Figure 1 presents the study concept and flowchart.

## Results

The three reviewers provided 1,170 ratings in total. The OQS, IS, and US were consistently high for all four LLMs in each subcategory. The overall intraclass correlation was good (ICC = 0.702). Specifically, CGT achieved the highest agreement (ICC = 0.765), followed by GA (ICC = 0.709), CS (ICC = 0.626), and ML (ICC = 0.599). This finding indicates a solid level of consistency in evaluating responses across different models, with CGT demonstrating the highest alignment with expert ratings. These results suggest varying reliability between models, with notable strengths in CGT and slightly lower consistency in ML.

### Subjective superiority

#### Radiological devices

For questions about energy consumption and energy-efficient technologies, the US consistently scored the highest score (US 4) across all four models. OQS performance was also very good for CGT and GA (OQS 4), with only ML (OQS 3.61 ± 0.5) and CS (OQS 3.89 ± 0.32) achieving moderately lower scores, reflecting strong quality and applicability of LLM advice on sustainability in radiology. CS (IS 4) and ML (IS 3.93 ± 0.26) scored the highest in IS, while CGT (IS 3.47 ± 0.52) and GA (IS 3.44 ± 0.53) offered solid, practical insights but achieved a slightly lower score. The MQS showed no significant differences between the LLMs in this subgroup.

#### Waste management

CS led in terms of OQS (4) and US (4), scoring consistently the highest. ML and GA scored lower, however, with overall good scores in all three categories (range: 3.44 ± 0.51 to 3.83 ± 0.38), showing particular strength in practical recommendations. CGT scored the lowest, particularly in IS (3.28 ± 0.46) and overall quality (3.22 ± 0.43), indicating that their responses were less actionable. The MQS reached by CGT was significantly lower compared to the other three LLMs (*p* < 0.001 to 0.008).

#### Digitalization

CS outperformed the other LLMs in this domain as well, achieving the highest scores across OQS (4), US (4), and MQS (3.91 ± 0.29). ML and GA performed very well in the US (GA 4; ML 3.89 ± 0.32), achieved marginally lower scores in IS (ML 3.78 ± 0.43; GA 3.94 ± 0.24) and OQS (ML 3.78 ± 0.43; GA 3.61 ± 0.5), and almost equal MQS (ML 3.82 ± 0.38; GA 3.85 ± 0.36). CGT showed the lowest scores in OQS (3.61 ± 0.5) and MQS (3.77 ± 0.42), struggling to articulate the nuances of digital transformation’s impact on sustainability. Despite these minor variations, the differences in the MQS across all LLMs were not statistically significant.

#### Practices and policies

CS was the top performer in this category, achieving the highest scores in OQS and IS (4) by providing structured and detailed frameworks. ML and CGT achieved equally slightly lower ratings in MQS (CGT 3.88 ± 0.32; ML 3.87 ± 0.34). GA performed very well in US and IS (3.83 ± 0.38) but showed lower results in OQS (3.06 ± 0.24; *p* < 0.001), resulting in a lower MQS (3.57 ± 0.5; *p* < 0.001).

#### Environmental impact

CGT achieved the highest scores across all three categories (4), resulting in the top MQS (4). CS received top scores in US and IS (4) with slightly lower OQS (3.94 ± 0.24) and MQS (3.98 ± 0.14). ML received consistently very good ratings in the US (3.94 ± 0.24) and IS (4), with slightly lower performance in OQS (3.83 ± 0.37), showing a limited grasp of this complex topic. GA offered solid practical measures but did not score as highly in comprehensiveness, with lower scores in OQS and consecutively in MQS (OQS 3.39 ± 0.5; US 3.89 ± 0.32; IS 4; MQS 3.75 ± 0.43). Compared to GA, the other three LLMs achieved significantly higher MQS (*p* < 0.001 to 0.018).

### Summary of results

Across all 30 questions on sustainability advice for radiologists, all four LLMs showed very good to good performance, with the highest ratings in understandability (CGT/ML/GA 3.91 ± 029; CS 3.99 ± 0.11), underlining the excellent language skills of the models. OQS (3.52–3.97) and IS (3.68–3.89) were also rated as good to very good among all LLMs, suggesting that practicability and usability meet expectations; they tend to show slightly lower scores in these categories compared to the US.

CS emerged as the top performer across most topics with MQS of 3.95 ± 0.22, frequently achieving the highest scores (*p* < 0.001), followed by ML (MQS 3.84 ± 0.37) and CGT (MQS 3.78 ± 0.42). GA received the lowest MQS (3.73 ± 0.44; *p* < 0.001 to 0.003).

The superior performance of CS can be attributed to its consistently clearer structure, radiology-specific contextualization, and more actionable recommendations, which led to higher US, OQS, and IS ratings and consequently higher MQS. Overall, the strong results across all models reflect their ability to synthesize established sustainability principles into practical advice for radiological workflows.

**Figure 2:**
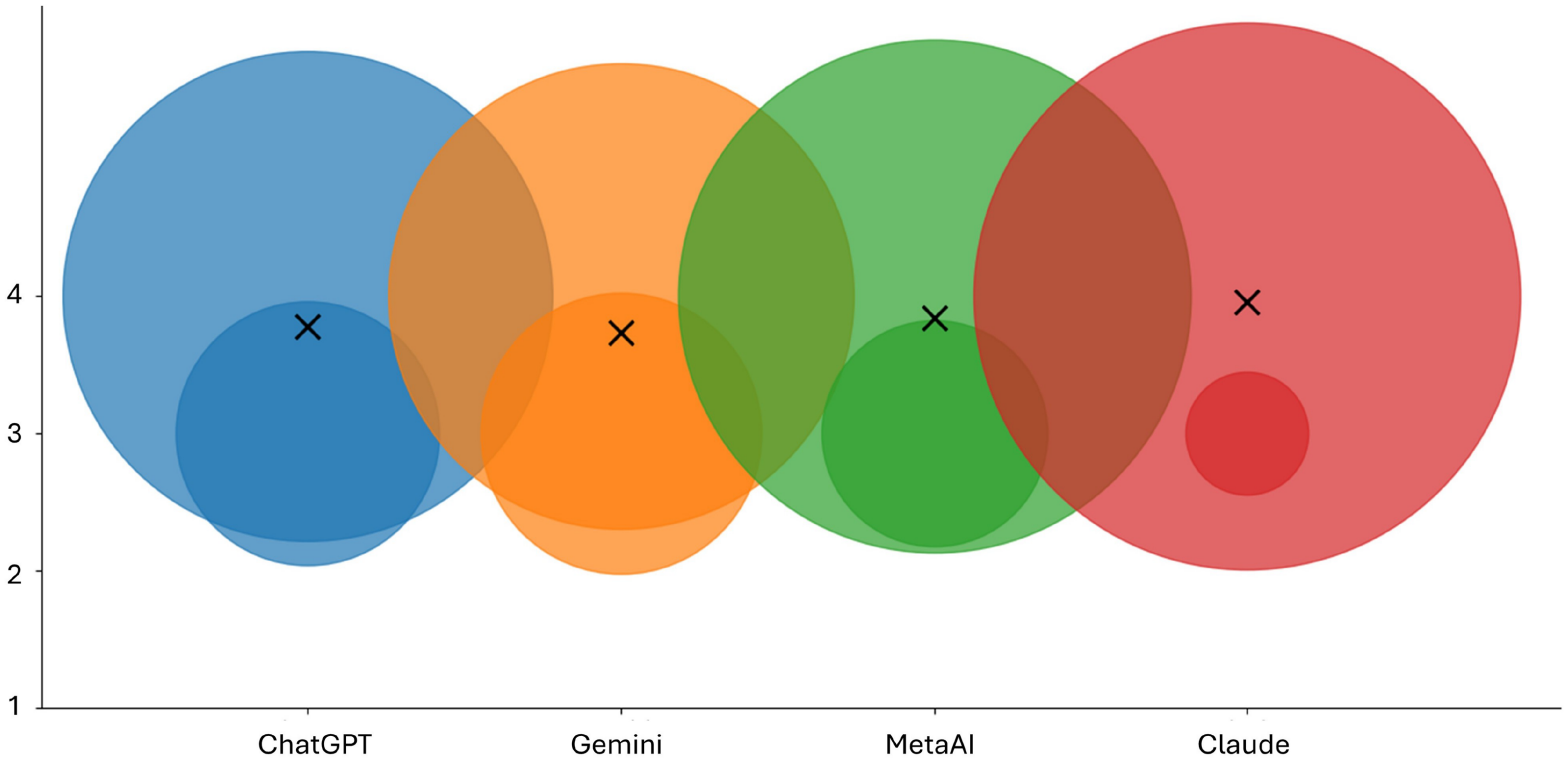
Distribution of ratings across the four evaluated LLMs. The vertical axis indicates the rating value, while the horizontal placement separates the models. Bubble chart presents the total number of ratings for answers provided by the four LLMs. The bubble sizes correlate with the rated scores for each chatbot. Crosses demonstrate the mean rated score for each LLM. This visualization highlights the overall performance level and variability of responses across models.

Table 1 and Figure 2 present a summary of the results.

**Table 1:** Comparison of ChatGPT-4.0 (ChatGPT/CGT), Gemini Advanced (Gemini/GA), Meta AI Llama 3.1 (Meta/ML), and Claude 3.5 Sonnet (Claude/CS) regarding overall quality score (OQS), understandability score (US) and implementability score (IS).

Topic	Scoring	ChatGPT	Gemini	Meta	Claude	Significant *p* values (range)
1	OQS	4	4	3.61 ± 0.5	3.89 ± 0.32	0.004
	US	4	4	4	4	–
	IS	3.47 ± 0.52	3.44 ± 0.53	3.93 ± 0.26	4	<0.001 to 0.017
	MQS	3.82 ± 0.37	3.81 ± 0.32	3.84 ± 0.37	3.96 ± 0.2	–
2	OQS	3.22 ± 0.43	3.56 ± 0.51	3.56 ± 0.51	4	<0.001 to 0.045
	US	3.61 ± 0.5	3.83 ± 0.38	3.83 ± 0.38	4	0.004
	IS	3.28 ± 0.46	3.44 ± 0.51	3.83 ± 0.38	3.72 ± 0.46	0.001 to 0.018
	MQS	3.37 ± 0.49	3.61 ± 0.49	3.74 ± 0.44	3.91 ± 0.29	<0.001 to 0.008
3	OQS	3.61 ± 0.5	3.61 ± 0.5	3.78 ± 0.43	4	0.004 to 0.039
	US	3.94 ± 0.24	4	3.89 ± 0.32	4	–
	IS	3.78 ± 0.43	3.94 ± 0.24	3.78 ± 0.43	3.72 ± 0.46	–
	MQS	3.77 ± 0.42	3.85 ± 0.36	3.82 ± 0.39	3.91 ± 0.29	–
4	OQS	3.83 ± 0.38	3.06 ± 0.24	3.83 ± 0.38	4	<0.001
	US	4	3.83 ± 0.38	3.89 ± 0.32	3.94 ± 0.24	–
	IS	3.83 ± 0.38	3.83 ± 0.38	3.89 ± 0.32	4	–
	MQS	3.88 ± 0.32	3.57 ± 0.5	3.87 ± 0.34	3.98 ± 0.14	<0.001
5	OQS	4	3.39 ± 0.5	3.83 ± 0.37	3.94 ± 0.24	<0.001 to 0.008
	US	4	3.89 ± 0.32	3.94 ± 0.24	4	–
	IS	4	4	4	4	–
	MQS	4	3.75 ± 0.43	3.93 ± 0.26	3.98 ± 0.14	<0.001 to 0.018
All	OQS	3.73 ± 0.45	3.52 ± 0.5	3.72 ± 0.45	3.97 ± 0.18	<0.001 to 0.006
	US	3.91 ± 0.29	3.91 ± 0.29	3.91 ± 0.29	3.99 ± 0.11	–
	IS	3.68 ± 0.47	3.77 ± 0.43	3.89 ± 0.32	3.89 ± 0.32	<0.001 to 0.041
	MQS	3.78 ± 0.42	3.73 ± 0.44	3.84 ± 0.37	3.95 ± 0.22	<0.001 to 0.003

In addition, the mean quality score (MQS) is presented. Each topic (1–5) is illustrated separately. Mean values with standard deviations (SD) are shown. The presented range of *p*-values indicates whether significant differences were observed between the LLMs. A “–” in the *p*-value fields denotes that no significant differences were found between the LLMs.

## Discussion

This study investigated the performance of LLMs in providing sustainability advice in radiology. Radiology accounts for a significant portion of a hospital’s overall energy consumption, highlighting an urgent need for more sustainable practices in this field (8, 9). In addition to the high energy demands and resource-intensive systems (e.g., helium-based technologies) (10), challenges such as waste management and material usage (e.g., the use of contrast agents) (13) underscore the necessity for improvements in sustainable practices within radiology. The importance of sustainability in radiology cannot be overstated, as the medical imaging field significantly contributes to environmental impact through those aspects. Therefore, integrating sustainable practices is essential to minimize radiology’s ecological footprint while maintaining high standards of patient care (7, 29, 30). A nationwide survey in Germany highlighted that 74.3% of radiology professionals consider sustainability important or very important in their work environment (7). However, only 38% of respondents reported that specific sustainability measures had been implemented at their institutions (7). This gap also underscores the need for actionable strategies and practical solutions to enhance sustainability in radiology. However, 16% of participants reported needing additional training and support due to insufficient knowledge to implement effectively sustainable practices (7).

In this study, LLMs were evaluated based on their overall quality, implementability, and understanding of sustainability measures in radiology. Overall, OQS, IS, and US scores were high for all four LLMs across all sustainability subcategories. The best ratings were consistently observed in understandability across all models, demonstrating uniformly strong performance. Beyond this, CS emerged as the best-performing model, dominating most categories with consistently high scores, followed by the other three LLMs. GA received slightly lower overall quality and IS ratings but performed well across all categories. As noted in previous studies (23, 25, 31), careful consideration of technical capabilities and LLMs’ performance is crucial for integrating AI tools in radiology later.

The performance of the tested LLMs in addressing sustainability aspects of radiology presents significant opportunities and essential challenges for the field. Our analysis demonstrates that current LLM technology has achieved a level of sophistication that enables meaningful contributions to sustainable radiological practices, similar to prior applications in other radiological subsections (32). The high quality scores across all evaluated models, particularly in understandability, align with the recent findings by Khanna et al. and suggest a promising foundation for clinical implementation (33). As previously emphasized, the environmental impact of radiological procedures necessitates innovative solutions, and LLMs appear well-positioned to contribute to this endeavor (34). Modest lower implementability scores indicate differences between a controlled setting and reality, as AI and LLMs have been shown to underperform in real-life environments in several different settings (35, 36).

However, our findings also reveal substantial variability in the intraclass correlation coefficients (ICCs) across the four LLMs evaluated, indicating that the quality and consistency of sustainability advice are highly model-dependent. While the overall results suggest that LLMs hold promise as tools for supporting sustainability initiatives, their robustness is not uniform. This variability highlights the importance of model selection when deploying LLMs for sustainability-related applications.

In our study design, we intentionally adopted a first-input scheme, as this approach most closely reflects real-world user behavior, where a single prompt is submitted and the initial response is used. While LLMs are known to exhibit some stochasticity in their outputs, our concept focused on evaluating the quality of first responses to align with practical application scenarios. Although we acknowledge that a broader analysis of output variability would offer additional insights, such an investigation was beyond the intended scope of this work. However, preliminary findings from our ongoing larger-scale studies suggest that newer LLMs show relatively low variability in their outputs. However, as Williams et al. highlighted, the complexity of clinical decision-making often extends beyond the capabilities of even advanced LLMs (37), leading to the need for further developments of LLMs (e.g., focused on different disciplines such as radiology).

Also, LLMs’ performance is inherently influenced by the limitations of their training data, which may not fully capture the diversity and complexity of radiological practices at the moment. While sustainability challenges in healthcare are well recognized, the application of LLMs to support sustainability efforts—particularly in medicine and radiology—is still a novel and evolving area. Looking ahead, longitudinal evaluations will be essential to monitor the consistency and reliability of LLM performance over time, as clinical practices and sustainability standards continue to develop. Future studies should therefore adopt a multidirectional approach, simultaneously addressing aspects such as technological integration, clinical validation, environmental impact, and cybersecurity. Establishing standardized frameworks for evaluating LLM contributions to medical sustainability—incorporating metrics like resource optimization and ecological footprint—would further strengthen the field. Moreover, integrating LLMs into existing radiological workflows, supported by robust validation studies, will be crucial to ensure efficiency gains are realized without compromising patient safety or data security.

Ultimately, these models show advanced possibilities driving forward sustainable directions in radiology, but evidence-based practices must guide implementation. Model selection should consider response quality, clarity, and implementability, as well as practical factors like accessibility, updates, transparency, and cost. Future research should explore the development of LLMs specifically trained on radiological sustainability data and prospectively evaluate their effectiveness in clinical workflows after implementation.

Overall, LLMs demonstrate significant potential as decision-support tools for implementing multidimensional sustainable practices in radiology, complementing clinical expertise to enhance resource efficiency while fostering new sustainable horizons in scientific research and clinical practice in radiology. However, such integration requires addressing regulatory frameworks, interoperability with existing hospital IT infrastructures, and validation in real-world clinical workflows (23, 25). While our findings highlight the potential of LLMs to provide high-quality, understandable, and implementable sustainability advice, translating these outputs into clinical decision-support tools would necessitate dedicated research efforts to ensure reliability, safety, and clinical acceptance. Additionally, the application of AI and LLMs in sustainability decision-making and clinical education raises important ethical considerations. These include questions of transparency, accountability, and potential biases in model outputs, as well as the responsibility of clinicians when relying on AI-generated advice (25). While addressing these issues in depth was beyond the scope of this study, future research and guidelines should explicitly incorporate such moral dimensions to ensure responsible and trustworthy integration of LLMs into radiology practice.

Our study has several limitations. First, we conducted a first-input study design. While this approach reflects a realistic real-world scenario, the use of multiple or varying inputs may influence the quality of LLM outputs. This potential variability warrants further investigation in future studies. Second, although we aimed to address the most relevant aspects of sustainability in radiology, some minor areas with comparatively lower impact may have been overlooked. Moreover, as the question set was derived from literature and expert consensus, selection bias in topic selection cannot be fully excluded. Another limitation of this study is that no direct comparison with human expert decision strategies in greening radiology was performed, which could provide valuable insights into complementarities and differences between human and LLM-based approaches. An additional limitation of this study is the omission of energy costs associated with digitalization, which is a contributing factor in the utilization of LLM for decision making in medical imaging. Further, the evaluation of effectiveness of these sustainable measures was beyond its scope and should be addressed in prospective implementation studies with dedicated study design. Future research should address these different dimensions to provide a more comprehensive evaluation of sustainability in radiology.

## Conclusion

Our findings demonstrate the potential of LLMs in advancing sustainability initiatives in radiology. The high performance of Claude 3.5 Sonnet, ChatGPT-4.0, Meta Llama 3.1, and Gemini Advanced across multiple dimensions suggests a promising future for LLM-assisted sustainable practices in radiology. However, careful consideration must be given to variabilities across the models and their responses, showing the importance of model selection when deploying LLMs for sustainability-related questions. Future research should explore the development of LLMs specifically trained on radiological sustainability data to address the field’s unique needs.

## Data Availability

The raw data supporting the conclusions of this article will be made available by the authors, without undue reservation.
