# Supplementary material for: Utilization of large language models in decision-making for sustainability in radiology
Source: Front Med (Lausanne). 2025 Sep 30;12:1632925. doi: 10.3389/fmed.2025.1632925 (PMC12518253; doi:10.3389/fmed.2025.1632925)
Supplement: Supplementary file 1 [file Table_1.DOCX]

**Questions**

**Topic 1: Radiological Devices**

- Which energy-efficient technologies can be integrated into existing radiology equipment to enhance sustainability?
- What are the most effective strategies for reducing energy consumption in radiology departments without compromising diagnostic quality?
- How can radiology departments optimize energy use across different operational modes of imaging devices (standby, active, idle)?
- What are the potential benefits and challenges of transitioning to greener imaging modalities (e.g., low-dose CT, ultrasound) over traditional ones?
- What role can energy monitoring and management systems play in improving the energy efficiency of radiological equipment?
- How does the energy consumption of radiological devices impact the overall energy footprint of healthcare facilities?

**Topic 2: Waste Management**

- What are the best practices for minimizing waste generation from disposable materials and packaging in radiology departments?
- How can radiology departments implement sustainable waste disposal methods, particularly for hazardous medical waste?
- Are there viable recycling or reuse programs for radiology-specific materials, such as film, plastics, contrast-agent, or electronic components?
- How can the use and disposal of contrast agents be optimized to minimize their environmental impact?
- How can the use of contrast agent be reduced?
- What sustainable alternatives to conventional contrast agents exist, and how can they be integrated into routine practice?

**Topic 3: Digitalization**

- How can digital technologies and teleradiology be leveraged to reduce the environmental footprint of radiology services?
- What are the environmental implications of increased digitization in radiology, and how can they be mitigated?
- What sustainable options are available for data storage, management, and archiving in radiology?
- What are the sustainability benefits of adopting teleradiology and how can these be measured?
- What emerging digital technologies can further contribute to sustainability in radiology?
- How can radiology departments stay informed about and implement sustainable digital innovations?

**Topic 4: Practices & Policies**

- What are the key components of a sustainable radiology department, and how can they be effectively implemented?
- Considering the topics 1. Energy consumption of radiological devices, 2. Waste management and contrast agent as well as Digitalization and Teleradiology - Which measures are most important to improve sustainability of radiology departments and how much can these reduce the environmental impact?
- What policies should be promoted at institutional and governmental levels to encourage sustainability in radiology?
- How can radiology departments lead or influence policy changes that support broader environmental sustainability in healthcare?
- What considerations are crucial when designing new radiology facilities with sustainability in mind?
- How can existing radiology facilities be retrofitted to enhance their environmental performance and sustainability?

**Topic 5: Environmental Impact**

- What practical steps can radiology departments take to reduce their carbon footprint and enhance sustainability?
- How can radiology departments effectively measure and track their carbon footprint and overall environmental impact?
- What initiatives, such as carbon offset programs, can radiology departments undertake to mitigate their environmental impact?
- How can the environmental impact of radiology be minimized through changes in operational practices and equipment use?
- How can radiology departments address the environmental impact of their supply chain, including the sourcing and disposal of materials?
- What long-term strategies can radiology departments adopt to ensure continuous improvement in their environmental sustainability?
